# Encephalitis Associated With Hemophagocytic Lymphohistiocytosis Secondary to Immune Checkpoint Inhibitors: An Unfamiliar Spin-Off

**DOI:** 10.7759/cureus.16079

**Published:** 2021-06-30

**Authors:** Ghulam Ghous, Hafiz Muhammad Hassan Shoukat, Zahid Ijaz Tarar, Muhammad Usman Zafar, Joseph W. McGreevy

**Affiliations:** 1 Internal Medicine, University of Missouri Columbia, Columbia, USA; 2 Internal Medicine, Premier Health/Wright State University, Dayton, USA; 3 Internal Medicine, University of Missouri, Columbia, USA; 4 Hospital Medicine, Lehigh Valley Health Network, Allentown, USA

**Keywords:** encephalitis, hlh, steroids, melanoma, ici

## Abstract

Checkpoint inhibitors (CPI) have become mainstream in standard therapy in various tumors, especially in malignant melanoma. Despite their widespread beneficial effects, these inhibitors are also notorious for immune-related adverse events (irAEs). Hemophagocytic lymphohistiocytosis (HLH) is an aggressive and life-threatening syndrome of excessive immune activation. We report a case of a 33-year-old male having a history of metastatic melanoma on immunotherapy (status post two cycles of ipilimumab/nivolumab) admitted for persistent fever and elevated liver enzymes. Additional work showed anemia, thrombocytopenia, hypertriglyceridemia, and hyperferritinemia which meet the diagnostic criteria of histiocyte society HLH-2004. The patient was effectively treated with oral prednisone. Moreover, further complications encompassed slurred speech, word-finding difficulties, ataxia, and lower extremity hyperreflexia concerning for autoimmune encephalitis. He was treated with high-dose IV methylprednisolone (1 gram/day for 3 days) with improvement in symptoms. Autoimmune encephalitis associated with HLH can be fatal - high-dose IV methylprednisolone should be considered, but this avenue still needs to be explored.

## Introduction

Immune checkpoint inhibitors (ICI), such as anti-programmed death-1 (PD-1) and anti-cytotoxic T-lymphocyte-associated antigen 4 (CTLA-4) antibodies, have shown widespread effectiveness in treating many cancers particularly malignant melanoma. On the downside, ICI therapy is associated with immune-related adverse events (irAEs) including pneumonitis, hepatitis, colitis, and endocrinopathies such as adrenal insufficiency, thyroid dysfunction, hypopituitarism, and type 1 diabetes mellitus [1]. However new and rare irAEs, including hematologic toxicities, are still an understudied territory, particularly in the case of dual ICI therapy. Management of hematologic irAEs such as thrombotic thrombocytopenic purpura/hemolytic uremic syndrome, aplastic anemia, autoimmune hemolytic anemia, and immune thrombocytopenia have been elaborated in recent literature [2]. Many case reports have been published lately describing hemophagocytic lymphohistiocytosis (HLH) in solid tumors secondary to ICI [3-9]. This case report is about a new finding of HLH associated encephalitis in a malignant melanoma patient treated with nivolumab/ipilimumab.

## Case presentation

A 33-year-old male having a history of malignant melanoma with metastatic disease to the bones on immunotherapy (status post 2 cycles of ipilimumab/nivolumab) admitted from the emergency department for persistent fever and elevated liver enzymes. The patient reported daily fever as high as 103.5° F (taken orally) for four days associated with two episodes of non-bloody emesis the day before admission. He had overall poor appetite but no abdominal pain or diarrhea. Headaches occurred only with fever. He denied any body aches, neck stiffness, photophobia. The patient was seen in urgent care three days ago with both negative rapid influenza and COVID-19 test along with no acute findings on chest X-ray. He was also seen in another hospital three days ago for the same symptoms, had blood cultures drawn, and discharged home on oral antibiotics. On physical examination, the patient had a temperature of 103° F, pulse 88 bpm, BP 134/81 mmHg, and oxygen saturation 96%. Respiratory, gastrointestinal, and neurological examinations were normal.

**Figure 1:**
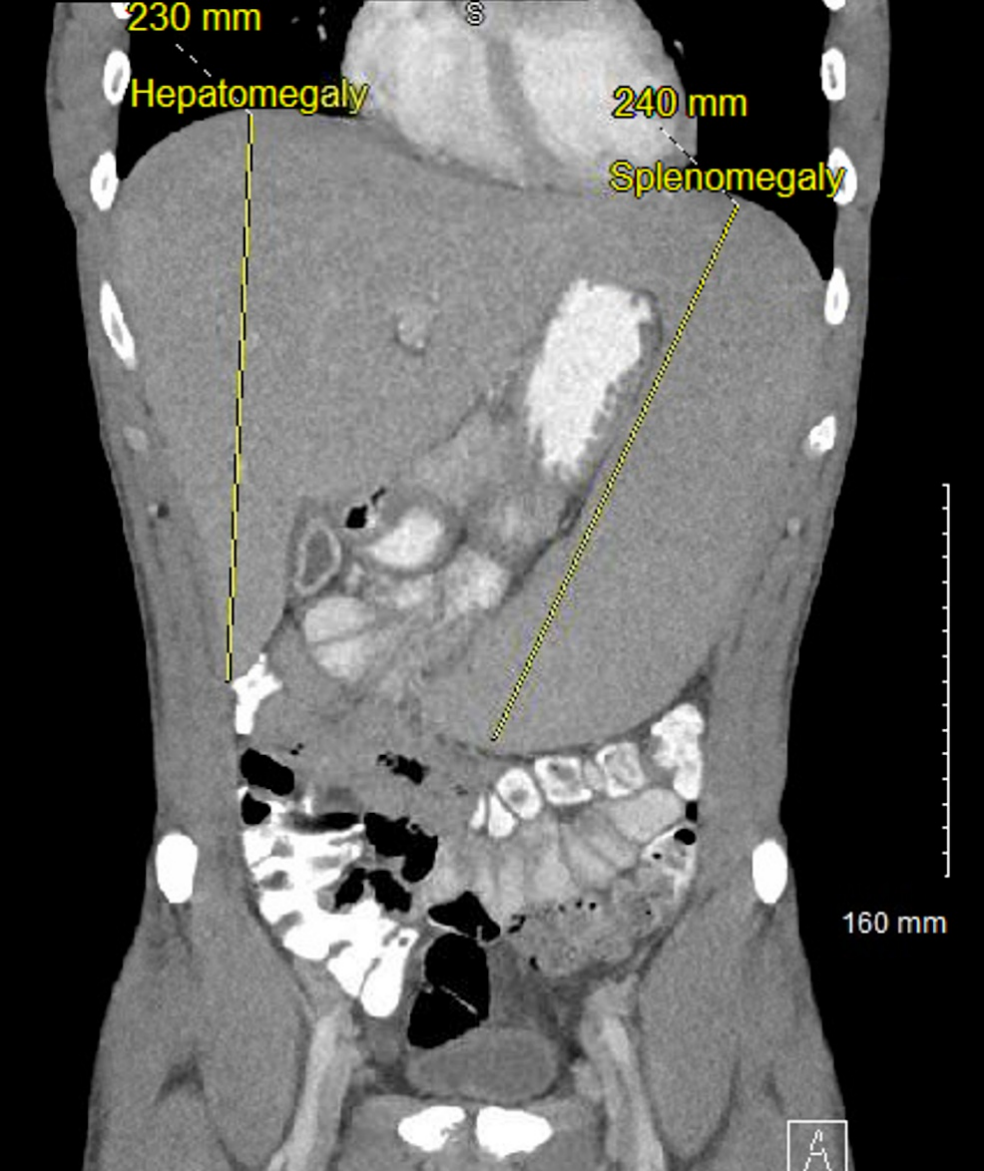
Contrast-enhanced CT abdomen and pelvis (coronal view) demonstrating hepatosplenomegaly.

**Figure 2:**
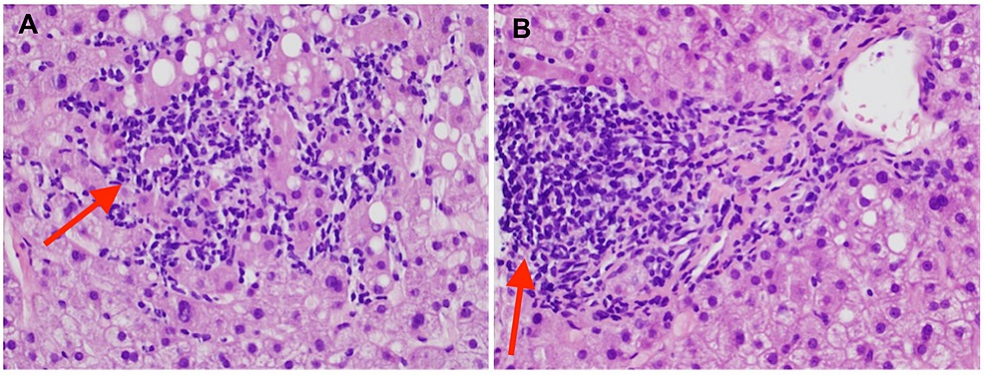
Photomicrographs of liver biopsy (H&E, 40x). (A) showing patchy lobular lymphocytic inflammation with mild macrovascular steatosis background. The presence of multifocal lobular lymphocytic inflammation (acute lobular hepatitis pattern of injury) is consistent with immunotherapy-associated liver toxicity. (B) Portal area showing focal moderate portal inflammation mixed with small mature lymphocytes and rare eosinophils suggestive of immune checkpoint inhibitors hepatotoxicity.

The initial investigations showed platelet count 94,000 per microliter (normal 150,000 to 400,000 per microliter), total bilirubin 3.56 mg/dl (normal 0.00- 1.60 mg/dl), alkaline phosphatase 409 units/L (normal 40-129 units/L), aspartate aminotransferase (AST) 316 units/L (normal less than 40 units/L), alanine transaminase (ALT) 363 units/L (normal 10-50). The right upper quadrant ultrasound demonstrated mild periportal edema, no evidence of gallstones or cholecystitis, non-dilated bile ducts. The patient was empirically started on piperacillin/tazobactam and vancomycin after sending two sets of blood cultures. Viral respiratory pathogen panel by polymerase chain reaction (PCR), autoimmune hepatitis work up, and viral hepatitis panel were also sent and all came back negative. The final blood culture report obtained from outside the hospital did not show any growth after four days. The patient continued to spike fever 101-103° F despite being on antibiotics for 24 hours. CT chest/abdomen/pelvis and MRI liver also obtained in the setting of persistent fever to look for occult infection and metastatic lesions in the liver but did not show any acute pathology other than hepatomegaly measuring 23 cm (normal less than 16 cm) and splenomegaly measuring 24 cm (normal less than 12 cm) (Figure 1). HIV, cytomegalovirus PCR, and Epstein-Barr virus PCR came negative. A hepatologist was consulted for elevated liver enzymes and recommended liver biopsy and prednisone 1mg/kg daily for possible immunotherapy-induced hepatitis. Liver biopsy showed multifocal lobular lymphocytic infiltrates within hepatic sinusoids (Figure 2A), and small mature lymphocytes long with rare eosinophils causing moderate portal inflammation, confirming immunotherapy-induced hepatitis (Figure 2B).

On day 4 of admission, the patient started to have slurred speech, word-finding difficulty, ataxia, and lower extremity hyperreflexia concerning for autoimmune encephalitis. Neurology was consulted and recommended paraneoplastic antibodies, MRI brain, and lumbar puncture. Serum ferritin, triglycerides, fibrinogen, and soluble IL-2 receptor (CD25) were also sent for possible HLH with associated encephalitis. Ferritin level came back 3446 ng/ml, triglycerides 525 mg/dl, fibrinogen 145 mg/dl, and soluble IL-2 receptor (CD25) 1405 pg/ml (age-related normal 1033 pg/ml). MRI brain with contrast showed no acute intracranial abnormalities. Serum ammonia level was also within normal limits. Cerebrospinal fluid (CSF) analysis showed elevated protein 199 mg/dl (normal 15-45 mg/dl) with normal WBC count. Oligoclonal bands in serum and CSF were 6 and the IgG index was 0.67 (normal 0.32-0.60). Paraneoplastic antibody panel (type 1 antineuronal nuclear antibody [ANNA-1], type 2 antineuronal nuclear antibody [ANNA-2], Purkinje cell cytoplasmic antibody type 1 [PCA-1], collapsin response-mediator protein-5 [CRMP-5] antibody, amphiphysin antibody,anti-N-methyl-D-aspartate [NMDA] receptor antibody, gamma-aminobutyric acid type A [GABA-A] and type B [GABA-B] receptor antibodies, contactin-associated protein-like 2 [Caspr2] antibody) was normal except for mildly elevated glutamic acid decarboxylase (GAD) antibody. GAD autoantibodies (GADAs) are associated with various neurologic conditions, such as stiff person syndrome, cerebellar ataxia, limbic encephalitis, myasthenia gravis, and epilepsy. Elevated GAD antibodies in our case were suggestive of encephalitis.

The patient was started on prednisone 1mg/kg daily for immunotherapy-induced hepatitis. Prednisone was increased to 2mg/kg on day 2 for worsening hyperbilirubinemia. The patient’s fever resolved 24 hours after starting steroids. Antibiotics were stopped (absence of infection). Liver enzymes also started to improve. Few days into admission, the patient started to develop neurological symptoms and after the aforementioned workup, he was diagnosed with encephalitis associated with HLH secondary to ipilimumab/nivolumab. Methylprednisolone was increased to 1 gram daily for three days with improvement in all neurological symptoms and the patient was discharged home on prednisone taper.

**Figure 3:**
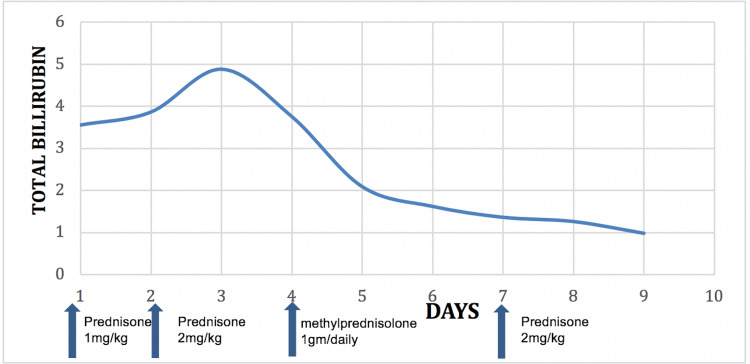
Total bilirubin levels in response to steroids.

**Figure 4:**
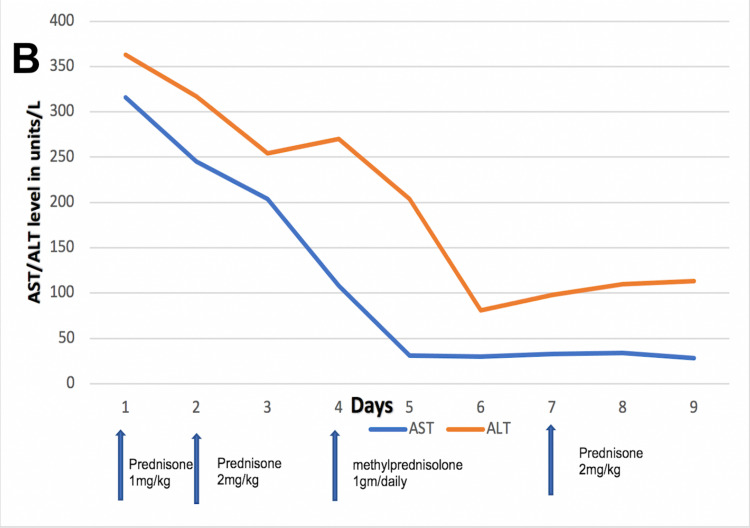
AST and ALT levels in response steroids. ALT, alanine transaminase; AST, aspartate aminotransferase.

The patient’s fever settled a day after starting steroids and all neurological symptoms improved with high-dose IV methylprednisolone (1 gram daily for three days). Liver enzymes also started to improve, and he was discharged on prednisone 2 mg/kg daily. AST and bilirubin normalized after 10 days (Figure 3 and Figure 4). ALT also started to improve but plateaued (Figure 4). The patient was advised to continue prednisone 2 mg/kg daily till ALT level gets orderly. At eight weeks follow-up, ALT normalized, and prednisone was gradually tapered over the next four weeks. The patient had no new neurological symptoms throughout treatment.

## Discussion

Hemophagocytic lymphohistiocytosis (HLH) is a progressively fatal syndrome of hyperactive immune system and requires prompt attention. Diagnostic criteria for HLH is listed below (Table 1) [3-5].

**Table 1:** HLH-2004 diagnostic criteria. ^a^Supportive criteria include neurologic symptoms, cerebrospinal pleocytosis, conjugated hyperbilirubinemia and transaminitis, hypoalbuminemia, hyponatremia, elevated D-dimers, and lactate dehydrogenase. The absence of hemophagocytosis (in the bone marrow) does not exclude a diagnosis of HLH ^b^New data show normal variation by age. Level should be compared with age-related norms. HLH, Hemophagocytic lymphohistiocytosis; NK-cell, Natural killer cells.

The diagnosis of HLH can be established if either 1 or 2 are fulfilled:
1. A molecular diagnosis consistent with HLH: pathological mutations of PRF1, UNC13D, STXBP1, RAB27A, STX11, SH2D1A, or XIAP
2. Diagnostic criteria for HLH fulfilled (5 out of the 8 criteria below): ^a^
Fever 38·5 °C (101.3 °F) or more
Splenomegaly
Cytopenias (affecting at least 2 of 3 cell lineages in the peripheral blood):
Hemoglobin
Platelets < 100 × 10^9^/L
Neutrophils < 1.0 × 10^9^/L
Platelets < 100 × 10^9^/L
Hypertriglyceridemia and/or hypofibrinogenemia
Fasting triglycerides ≥3.0 mmol/L (i.e., ≥ 265 mg/dL)
Fibrinogen ≤1.5 g/L
Hemophagocytosis in bone marrow or spleen or lymph nodes or liver
Low or absent NK-cell activity
Ferritin ≥500 mg/L
Soluble CD25 (i.e., soluble IL-2 receptor) ≥ 2400 U/mL^b^

North American Consortium for Histiocytosis (NACHO) advocate against the terms "primary HLH" and "secondary HLH," owing to the fact that these terms cause confusion as both primary and secondary HLH can be activated by infections or other immune-related pathologies, and gene mutations can occur in any person regardless of age and family history [6]. NACHO prefers the following terminologies [6]:

HLH syndrome - A condition of pathologic immune activation that is often associated with genetic defects of lymphocyte cytotoxicity. This includes all conditions meeting consensus diagnostic criteria HLH disease - HLH syndrome in which the distinctive immune activation is the core problem and that would benefit from HLH-directed immunosuppressive therapies such as HLH observed after immune-activating therapies (iatrogenic HLH, also called cytokine release syndrome), familial HLH with clear genetic etiology, HLH associated with malignancy, HLH associated with rheumatologic conditions (also called MAS).

HLH disease mimics - Other disorders that resemble HLH syndrome but are caused by other conditions and that would not benefit from immunosuppressive therapy or require entirely different treatments.

Pathophysiology of HLH comprises abnormal working of macrophages, natural killer cells (NK cells), cytotoxic lymphocytes (CTLs), and altered numbers of CD4 and CD8 lymphocyte subsets [7]. HLH induces organ failure because of tissue damage as an outcome of over activation of macrophages resulting in over secretion of cytokines. CTLs or NK cells are not able to remove activated macrophages leading to enhanced activity of macrophages and raised values of various cytokines such as interferon-gamma. HLH patients have diminished cytotoxic activity of CTLs and NK cells, associated with enhanced macrophage activation. This stimulated production of cytokines is considered a major perpetrator of tissue injury and multi-organ failure leading to high mortality [8-10]. 

The onset of an acute episode of HLH can trigger because of infection or modification in immune balance which can occur either due to immune activation or immune deficiency. Immune activation from an infection is a common trigger [11]. The immune checkpoint inhibitors can also cause immune activation and HLH, but the incidence has not been defined.

We found a total of 10 cases of ICI-associated HLH in malignant melanoma including nivolumab [12], pembrolizumab [5,13], combined pembrolizumab, and ipilimumab [14], combined ipilimumab and nivolumab [12,14-16]. Six of ten patients had no other immune-related toxicities while 4 others showed thyroiditis [16], hepatic cytolysis, lymphocytic meningitis, and colitis [14]. All 10 patients received steroids in the 0.5 to 2mg/kg range. Out of those, three were given extra immunosuppression, one with mycophenolate mofetil [16], and one with etoposide and tocilizumab [14], and the other with etoposide [14] (Table 2).

**Table 2:** Summary of hemophagocytic lymphohistiocytosis cases in malignant melanoma secondary to immune checkpoint inhibitors therapy. HLH: Hemophagocytic lymphohistiocytosis

Author/year	Age	Immunotherapy	Time/ cycles of therapy	Other immune-related toxicities	Treatment	Clinical outcome
Satzger et al [16]. 2018	F/ 26	Ipilimumab and nivolumab	4 doses	Thyroiditis, hepatitis	Prednisone 2 mg/kg/d mycophenolate mofetil 360 × 2 then 720 mg × 2/d	Resolution of HLH
Hantel et al [15]. 2018	F/ 35	Ipilimumab and nivolumab	4 doses of ipilimumab, 1 dose of ipilimumab and nivolumab (3 weeks prior)	None	Methylprednisolone 1.5 mg/kg/d × 3 for 4d Then prednisone 1 mg/kg	Resolution of HLH
Malissen et al [12]. 2017	M/ 42	Ipilimumab and nivolumab	1 dose of ipilimumab; prior history of 9 months of nivolumab. symptoms 5 days after ipilimumab	None	Systemic corticosteroid treatment	Resolution of HLH
Malissen et al [12]. 2017	M/ 77	Nivolumab	17 months	None	Prednisone 0.5 mg/kg/d	Passed away after 6 weeks
Sadaat et al [5]. 2018	M/ 58	Pembrolizumab	6 doses	None	Prednisone 1 mg/kg/d	Resolution of HLH
Sasaki et al [13]. 2018	F/ 60	Pembrolizumab then dabrafenib and trametinib due to progression	7 doses	None	Prednisone 0.5 mg/kg	Resolution of HLH
Dupre et al [14]. 2020	F/ 35	Ipilimumab and nivolumab	1 dose	None	Corticosteroids 500 mg IV then PO etoposide 150 mg/m^2^ IV Then IV Ig 1 g/kg, then tocilizumab	Partial control of HLH
Dupre et al [14]. 2020	F/ 52	Pembrolizumab 8 cycles then ipilimumab 2 cycles	28 days after ipilimumab	Hepatitis	Prednisone 1 mg/kg/d etoposide 150 mg/m^2^ IV	Dead from HLH with cerebral hemorrhage
Dupre et al [14]. 2020	M/ 69	Nivolumab 2cycles then ipilimumab 2 cycles	5 weeks after ipilimumab	Hepatic cytolysis and lymphocytic meningitis	Corticosteroids 1 mg/kg/d	Resolution of HLH
Dupre et al [14]. 2020	M/ 27	Pembrolizumab 3 cycles then nivolumab + ipilimumab 2 cycles	4 weeks after nivolumab + ipilimumab	Hypophysitis, lymphocytic meningitis, colitis, hepatic cytolysis	Methylprednisolone 100 mg/d	Resolution of HLH
Our Case	M/ 33	Ipilimumab and nivolumab 2 cycles	4 weeks after nivolumab + ipilimumab	Hepatitis, encephalitis	Prednisone 2 mg/kg/d then methylprednisolone 1 gram daily for 3 days followed by oral prednisone taper	Resolution of HLH, encephalitis and hepatitis

We report the first case of encephalitis associated with HLH in the setting of ICI for malignant melanoma. Initially, we treated the patient with prednisone 1mg/kg for HLH but when he showed symptoms of encephalitis including slurred speech, word-finding difficulties, ataxia, and lower extremity hyperreflexia we increased steroids to methylprednisolone to 1 gram daily and treated the patient for three days with complete resolution of encephalitis symptoms.

## Conclusions

This is the first reported case of encephalitis associated with HLH secondary to combination ICI therapy in the setting of malignant melanoma treated with high dose methylprednisolone (1gm/daily for 3 days). HLH-associated encephalitis should be suspected in a patient exhibiting neurological symptoms other than HLH features, high-dose IV methylprednisolone 1gm/daily should be considered but further evidence is required.
